# Safety of Hyaluronan 35 in Healthy Human Subjects: A Pilot Study

**DOI:** 10.3390/nu11051135

**Published:** 2019-05-22

**Authors:** Annette Bellar, Sean P. Kessler, Dana R. Obery, Naseer Sangwan, Nicole Welch, Laura E. Nagy, Srinivasan Dasarathy, Carol de la Motte

**Affiliations:** 1 Department of Inflammation and Immunity, Lerner Research Institute, Cleveland Clinic, Cleveland, OH 44195, USA; bellara@ccf.org (A.B.), kessles@ccf.org (S.P.K.), oberyd@ccf.org (D.R.O.); welchn@ccf.org (N.W.); nagyl3@ccf.org (L.E.N.); 2 Center for Microbiome and Human Health, Lerner Research Institute, Cleveland Clinic, Cleveland, OH 44195, USA; sangwan@ccf.org

**Keywords:** hyaluronan, anti-inflammatory, clinical trial, human, microbiome

## Abstract

*Background*. Hyaluronan (HA) is a naturally occurring glycosaminoglycan polymer produced in all vertebrates, and usually present at the high molecular weight (>10^6^ Da). Low molecular weight HA has signaling properties, and fragments ~35 kDa size (HA35) have biological activity in eliciting epithelial β-defensins and tight junction proteins, notably ZO1, important components of innate host defense arsenal of the gut barrier in preclinical models. Safety, tolerability, impact on metabolism, gut permeability, and microbiome composition in healthy human subjects were all evaluated prospectively. *Methods*. Pharmaceutical grade HA35 (140 mg in water once daily for seven days), was administered orally to 20 healthy subjects (30.7 ± 5.6 years). Demographical, clinical, biochemical laboratory tests, metabolic function and stool microbiome composition were measured on Day 0, 8 and 28. *Results*. HA35 was tolerated well in all subjects with no serious adverse events in any subjects. No statistical differences in any of the measurements were seen among the study group over the course of the trial. In aggregate there were no changes in demographical, clinical, biochemical laboratory tests, and metabolic function or microbiome composition during the 28-day study. *Conclusion*. Oral HA35 administration (140 mg/day) is a safe treatment in healthy individuals and does not affect metabolic, inflammatory or microbiome parameters.

## 1. Introduction.

Hyaluronan (HA) is a linear glycosaminoglycan polymer consisting of repeating disaccharides of β-glucuronic acid and N-acetylglucosamine [1]. Physiologically, HA is synthesized by hyaluronan synthetases as high molecular weight polymers of up to 10,000 KDa and is a major component of the extracellular matrix [2]. Size-specific functions of HA have been reported and in general larger polymers of HA promote homeostasis while smaller size HA fragments have been shown to signal intracellular pathways in cells [3,4,5]. HA is synthesized by Class I HA synthetases (HAS) that are lipid-dependent membrane proteins and mammals express three HAS isozymes coded by HAS1, HAS2 and HAS3 genes [6]. Of these, HAS3 is induced by IL1β in vascular smooth muscle cells and involved in macrophage driven inflammation and atherogenic plaque progression [7] and tumor growth [8]. Degradation of large HA polymers into smaller sized fragments occurs during inflammation and processes of natural turnover [3,5].

The principal effects of HA are mediated through HA binding to its receptors, including CD44, a major plasma membrane receptor expressed in many types of cells, including leukocytes, epithelial, smooth muscle cells, fibroblasts and endothelial cells. Additionally, toll-like receptors (TLRs) 2 and 4, receptor for HA-mediated motility (RHAMM), lymphatic vessel endothelial HA receptor 1 (LYVE1), HA receptor for endocytosis (HARE; Stab2) and Layilin are receptors through which HA binds and signals [1,9,10,11,12,13]. While LYVE-1 and HARE are mostly restricted to lymphatic vessels and liver sinusoids, the other receptors are widely expressed in many cell types.

Recent studies have shown that exogenous, specific-sized HA of an average molecular weight of 35 Kda (HA35), but not smaller or larger polymers, dampens inflammatory responses in murine models of inflammation, including bacterial-driven colitis and alcohol-mediated injury [14,15,16,17]. These beneficial effects are mediated by HA35 interaction with multiple HA receptors, including TLR 4, CD44 and Layilin expressed in intestinal epithelial cells and Kupffer cells [18]. We have also previously reported that oral administration of HA35 to healthy wild type mice increases the expression of intestinal β-defensins 3 and 4 through a TLR-4 mediated pathway and protects from both *Salmonella* Typhimurium and *Citobacter rodentium* infections [14,18]. Adding to the innate barrier defenses, oral HA35 supplementation increases tight junction protein zona occluden-1 (ZO-1) in the distal colon epithelium during challenge with *Citrobacter* and dextran sodium sulfate (DSS), as well as in isolated intestinal organoids [14,16]. Orally provided HA35 also decreases the pore forming or ‘leaky’ claudin 2 induced in the epithelium in an in vivo *Salmonella* infection model [18]. In keeping with tight junction regulation, HA35 decreases intestinal permeability in the damaged intestine [16]. Additionally, HA35 normalizes the exacerbated Kupffer cell inflammatory response to lipopolysaccharide (LPS) induced by alcohol in vitro and lessens disease severity in alcohol-fed animal models [15,17]. In rodents, the gut microbiome was also altered by HA35 (C de la Motte and SPK, unpublished data), but whether such changes occur in humans and if they are reversible after stopping HA35 supplementation are not known.

These preclinical data suggest the direct potential for therapeutic efficacy in protecting against conditions that are driven by a dysregulated gut barrier and bacterial translocation, such as colitis and alcoholic liver disease. Therefore, as a first step, we evaluated the safety and tolerability of orally administered HA35 in healthy human subjects using a non-invasive measure of intestinal permeability and systemic cytokine responses. Since alterations in intestinal responses, including inflammatory cell infiltration quantified by fecal calprotectin, a neutrophil protein that has been used as a measure of gut inflammation, and measurement of fecal β-defensin-2 (human homologue of mouse β-defensin 3), an endogenous naturally occurring antibiotic peptide that is a part of the intestinal epithelial barrier defense system [18,19,20]. The fecal microbiome was evaluated before and after HA35 supplementation. Whole body metabolic responses and substrate utilization were quantified using indirect calorimetry and biochemical assays used to assess liver and renal function. Fecal microbiome analysis was performed on samples prior to, immediately following and post-treatment to assess changes in the intestinal bacterial environment. Symptoms during HA35 supplementation and following the end of the study were also documented. These studies are of high clinical relevance, due to the availability of pharmaceutical grade HA35 that has the potential human use in targeting tissue inflammation.

## 2. Patients and Methods

### Study Subjects

**Figure 1:**
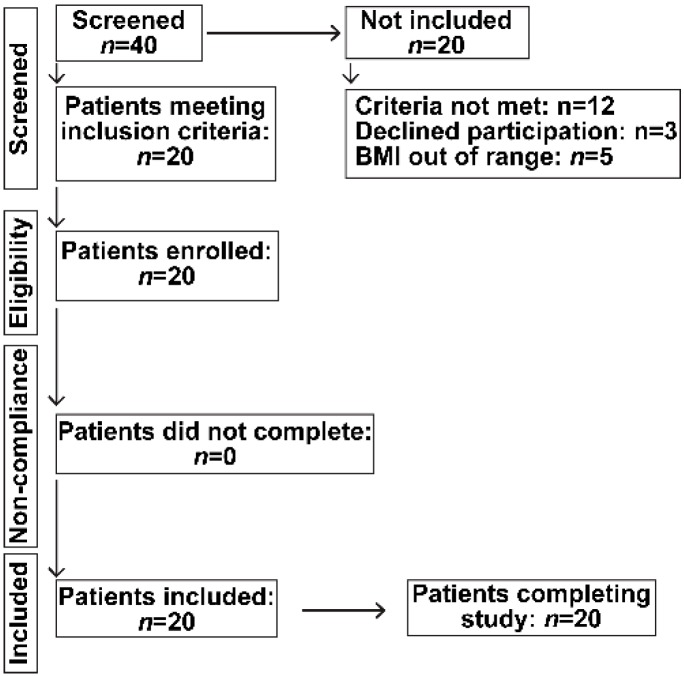
Flow chart of recruitment of participants according to the Consort statement.

Healthy, non-hospitalized, human subjects of both genders were screened for inclusion in a prospective study of oral HA35 once daily for seven days after which they were followed for a total of 28 days. Only young adults between age 18–45 years were included, since ageing may affect intestinal immunity, microbiome composition and the presence of underlying illnesses. Subjects who were pregnant, nursing, incarcerated or had documented diabetes mellitus, hypertension, chronic organ dysfunction, including renal, heart, liver or lung disease, malignancy or on chronic prescription medication were excluded. Of the 22 patients who satisfied the inclusion and exclusion criteria, 20 were included and all subjects completed the study undertaken in accordance with the CONSORT guidelines (Figure 1).

The screening visit included informed consent, medical history, physical examination, diet history (including probiotics and food allergies) using the Diet History Questionnaire II [21], and laboratory testing. Patients were asked to refrain from chronic NSAIDs (i.e., more frequent dosing than twice per week), gastric acid suppressants, new systemic (oral, intravenous) and local enteric (laxatives, enemas) medications. All patients were on their usual home diet with no specific dietary recommendations and were asked to continue with their usual levels of physical activity. No patients received enteral or parenteral nutrition.

Oral HA35 (140 mg) was dissolved in 20 mL of filtered, sterile, unflavored water and dispensed into aseptic plastic tubes, prepared in a sterile hood. Each patient was provided with seven doses of HA35 for oral ingestion at home. Subjects ingested HA35 once daily, 30 min before breakfast. The initial dosage was chosen from the functional dose we determined in mice and using intestinal surface area ratio calculations [14] scaled to an average of 70 kg. human. Double the calculated dose was dispensed to account for the maximal potential weight of a study participant. The dosage derived by this method is slightly higher, but still in general agreement with, the calculated dose recommended by body surface area conversion methods. All subjects were provided detailed instructions on how to store their supplement under refrigeration and on how to take the supplement orally. Compliance was monitored by regular phone calls and confirmed by the study coordinator verbally upon return for the D8 visit. Study subjects kept a daily log of gastrointestinal symptoms, a food diary and a stool frequency chart. On D0 (prior to the start of HA35), D8 and D28 subjects provided a stool sample from the entire day (±24 h). Stool samples were refrigerated immediately after collection, samples were homogenized and frozen in aliquots at −80 °C until the study was completed All samples were processed at the same time for microbiome analysis and antimicrobial peptide content quantification. Venous blood was obtained for separation of serum on D0, D8, and D28 in the Clinical Research Unit (CRU) at the Cleveland Clinic. Samples were stored at −80 °C for future analyses.

All study activities were approved by the Cleveland Clinic Institutional Review Board (IRB) after obtaining written informed consent and were in conformity with the Helsinki Declaration of Human Rights. It was undertaken in accordance with the CONSORT guidelines (Figure 1). The study was registered in ClinicalTrials.gov (Trial registration. NCT 02867605).

Commercially available pharmaceutical grade sodium hyaluronate fragments of 35 KD (Lifecore Biomedical LLC, Chaska, MN, USA) was used in these studies. Per the manufacturer, Lifecore’s sodium hyaluronate is produced by an efficient microbial fermentation and purified by a highly effective process, it ensures consistency in supply. Regulatory approval has been obtained by Lifecore, including the certificate of suitability (CEP) to the Sodium Hyaluronate Monograph in the European Pharmacopeia and complies with EU Pharmacopoeia monograph for NaHy, compliance with Japanese pharmacopoeia, ISO13485 certified quality system, ICH A7 (Good Manufacturing Practice Guide for Active Pharmaceutical Ingredients) compliance, CE Mark Certification on Lifecore’s Hyaluronan Based Products, Drug and Device Master Files, GMP certification and 21 CFR 210, 211, 820 and drug and device compliance.

## 3. Methods

### 3.1. Clinical Assessment

Anthropometric measurements (whole body weight, height) were obtained in a standard hospital gown on a calibrated scale and a wall-mounted stadiometer. Body mass index (BMI) was calculated as body mass (kg) divided by height (m^2^). Brachial blood pressure was obtained in all subjects on the left arm under standard, quiet conditions in the clinical research unit after at least 5 min of rest.

### 3.2. Clinical Laboratory Tests

After an overnight fast, blood glucose, serum amino transferases, blood urea nitrogen, serum creatinine, albumin and bilirubin were obtained on the day of the study in the Clinical Research Unit laboratory. Samples were drawn after an overnight fast to limit the impact of variability in the time, quantity and type of food intake on the laboratory results if patients were allowed unrestricted dietary intake.

### 3.3. Indirect Calorimetry

The rate of oxygen consumption (VO_2_ )and the rate of carbon dioxide production (VCO_2_) were quantified using an open canopy indirect calorimeter (Vmax Encore, Viasys, Yorba Linda, CA, USA) in the CRU, as previously described [22]. In brief, patients were allowed to rest for 10 min prior to the determination of substrate oxidation and energy expenditure. Breath samples were collected for a total of 15 min, and only the last 5 min were used for analysis. The calorimeter was calibrated prior to each study and was carefully checked for any leaks.

### 3.4. Stool Microbiome Diversity

Stool samples were collected on the first day prior to the start (D0), the last day of (D8) and D28 of the start of HA35 supplement to determine if any changes in the microbiome were observed during or after stopping HA35. There is limited data on the impact of a short duration of overnight fasting as in this study prior to sample collection and we chose this to limit the impact of variability in dietary intake on the microbiome [23]. *Sample preparation*. Composition of stool microbiome was measured by 16S pyrosequencing conducted by Second Genome (San Francisco, CA, USA). Nucleic acids were isolated using a MoBio PowerMag microbiome kit (Carlsbad, CA, USA) according to the manufacturer’s instructions and samples quantified via the Qubit Quant-IT dsDNA high sensitivity Kit (Invitrogen, Life Technologies, Grand Island, NY) to ensure minimum concentration and mass of DNA. *Library preparation*. Bacterial 16S V4 rDNA region was amplified using fusion primers designed for the surrounding conserved regions tailed with sequences to incorporate Illumina (San Diego, CA, USA) adapters and indexing barcodes. Each sample was PCR amplified with two differently bar coded V4 fusion primers and the PCR products quantified. Samples that met the post PCR quantification minimum were pooled in equimolar quantities and sequences. *Profiling method*. The pool of 16S V4 enriched, amplified, barcoded samples were loaded on a MiSeq reagent cartridge and then into the instrument. After cluster formation on the MiSeq instrument, the amplicons were sequenced for 250 cycles with custom primers designed for paired end sequencing. *Bioinformatic analysis*. Sequenced paired-end reads were quality filtered and merged using USEARCH [24] and the resulting sequences were compared to the SecondGenome strains database using USEARCH (usearch_global). All sequences hitting a unique strain with an identity ≥99% were assigned a strain Operation Taxonomic Unit (OTU). To ensure specificity of the strain hits, a difference of ≥0.25% between the identity of the best hit and the second-best hit was required (e.g., 99.75 versus 99.5). For each strain OTU one of the matching reads was selected as representative and all sequences were mapped by USEARCH (usearch_global) against the strain OTU representatives to calculate strain abundances. The remaining non-strain sequences were quality filtered and dereplicated with USEARCH. The resulting unique sequences were then clustered at 97% by UPARSE (de novo OTU clustering) and a representative consensus sequence per de novo OTU was determined. The UPARSE clustering algorithm comprises a chimera filtering and discards likely chimeric OTUs [25]. All non-strain sequences that passed the quality filtering were mapped to the representative consensus sequences to generate an abundance table for de novo OTUs. Representative OTU sequences were assigned taxonomic classification via the mothur Bayesian classifier [26], trained against the Greengenes reference database [27] of 16S rRNA gene sequences clustered at 99%. The diversity function from the vegan package was used to calculate the alpha and diversity across the different set of samples (timepoints). Briefly, Shannon diversity and Bray-Curtis dissimilarity index were used to compare the alpha and abundance weighted beta-diversity patterns across all the test groups (i.e., Day 0, Day 8 and Day 28), respectively. Principal Coordinate Analysis (PCoA) was used to reduce the dimensionality of the microbiome data and the summary of the beta diversity relationships were visualized in two dimensional scatterplots implemented in the ggplot2 package in R [28]. Since the frequencies of some of the phyla were very low, they were combined under the category of others (p_Cyanobacteria; p_Lentisphaerae; p_Fusobacteria; p_Synergistetes) and unclassified were those species that could not be classified at any level.

Statistical analysis was done by Permutational Analysis of Variance (PERMANOVA) using the Benjamini-Hochberg correction for false discovery rate to compare categorical and continuous variables using Second Genome’s analysis software package. Univariate differential abundance of OTUs is tested using a negative binomial noise model for the over dispersion and Poisson process intrinsic to this data, as implemented in the DESeq2 package [29], and described for microbiome applications [30]. It considers both technical and biological variability between experimental conditions. DESeq was run under default settings and q-values were calculated with the Benjamini-Hochberg procedure to correct *p*-values, controlling for false discovery rates. The principal coordinate analysis was performed for 2-dimensional ordination plotting to visualize complex relationships between samples. Dendrogams were generated using the ward method for hierarchical clustering of distance matrices. All sequencing data were uploaded to PRJEB32537.

### 3.5. Serum Cytokines

To determine the effects of oral HA35 on systemic inflammation, human serum high-sensitivity C-reactive protein (RayBioTech, Norcross, GA, USA), tumor necrosis factor-α and interleukin-6 (BioLegend, San Diego, CA, USA), were quantified using commercially available ELISA kits per manufacturer recommendation.

### 3.6. Serum HA Levels

To determine whether oral HA35 affected circulating HA, total serum levels of HA were quantified using a highly sensitive, competitive ELISA-like assay according to the manufacturer’s protocol (Echelon Biosciences Inc., Salt Lake City, UT, USA).

### 3.7. Intestinal Permeability

Serum lipopolysaccharide was used as a measure of changes in intestinal permeability and quantified using an ELISA (MyBioSource, Inc., San Diego, CA, USA).

### 3.8. Antimicrobial Peptides in Stool

Calprotectin, an antimicrobial protein and neutrophil product that is released into the intestinal lumen during inflammation was assayed from the collected stool samples. Briefly, stool samples were incubated with phosphate buffered saline and agitated at 4C, before filtering. Protein determination of each sample was made using a Bradford assay and the results used to normalize the results. The filtrate was assayed using a calprotectin ELISA. (Affinity Diagnostics Corp., Toronto, ON, Canada).

Since HA35 increases β -defensin production in murine preclinical models [18], stool content of human β-defensin 2 (orthologue of the regulated mouse defensin) was measured. Using fecal samples processed as above, β -defensin was quantified by ELISA per manufacturer protocol (Affinity Diagnostics Corp., Toronto, ON, Canada).

### 3.9. Statistical Analyses

All data were expressed as mean ± standard deviation unless specified. Qualitative variables were compared using the Chi square test and quantitative variables compared using repeated measures ANOVA. Correlations were determined using the Pearson’s correlation coefficient. A *p* value < 0.05 was considered statistically significant. The sample size was estimated based on recruitment considerations and previous recommendations (Julious 2004) of a minimum sample size of 12 subjects based on precision about the mean and the variance. All analyses were performed using SPSS 20.0 (IBM, Armonk, NY, USA). Permutational Analysis of Variance (PERMANOVA) was utilized for finding significant differences among discrete categorical or continuous variables in the microbiome data. Univariate differential abundance of OTUs was tested using a negative binomial noise model for the over dispersion and Poisson process intrinsic to this data, as implemented in the DESeq2 package [29], and described for microbiome applications [30]. DESeq was run under default settings and *q*-values were calculated with the Benjamini-Hochberg procedure to correct *p*-values, controlling for false discovery rates.

## 4. Results

### 4.1. Clinical and Demographics

The mean age of the subjects in the study was 30.7 ± 5.6 years (range 22–43 years), including nine males and 11 females. Their ethnic distribution was as follows—15 Caucasians, 3 African American and 2 Asian. All subjects self-reported at least college level of education. All subjects completed the duration of supplementation. There were no changes in anthropometric or clinical evaluations between D0 and D8, during HA35 supplementation, or on D28, at the end of the follow-up period (Table 1). None of the subjects reported any serious adverse events during the ingestion of the supplement or at follow-up. Trial registration. NCT 02867605.

### 4.2. Clinical Laboratory Parameters

The clinical laboratory data at entry and follow-up are shown in Table 2. As expected, since only healthy subjects were included, all values were within normal ranges at entry. Oral HA35 for seven days did not cause any changes in blood counts, renal function or hepatic transaminases or other serum chemistries during supplementation with HA35 or follow-up after stopping the supplement.

### 4.3. Indirect Calorimetry Data

Measured resting energy expenditure and respiratory quotient are shown in Table 3 as a measure of whole-body substrate utilization at entry and following completion of the supplement. Measured REE as a percentage of the estimated REE by the standard Harris Benedict equation was also calculated. No statistically significant changes were found in any of these parameters during or following HA35 supplementation.

### 4.4. Serum Indicators of Inflammation and Injury

Circulating markers of inflammation, including high sensitivity CRP, TNFα and IL6, were quantified at all three time points during the study (Table 4) and showed no difference in response to oral HA35 before and at the end of treatment and when subjects were followed up to 28 days after 7 days of HA35.

### 4.5. Fecal Peptides: Calprotectin and β-Defensin 2

We also measured fecal antimicrobial peptide secretion in the patients at each study visit. No changes in total soluble fecal protein, normalized calprotectin or normalized β-defensin 2 were evident over the 28-day follow-up period (Table 5).

### 4.6. Serum levels of HA and Intestinal Permeability

Low levels of HA are normally present in circulation while elevated levels are proposed to be a marker of some diseases, including cirrhosis [31]. To determine whether oral HA35 affects the circulating levels of HA serum samples from D0, D8 and D28 were compared. No statistical change was observed in serum HA levels at any of these time points (Table 6). In addition to intestinal inflammation, we also quantified serum LPS concentration as an indirect measure of intestinal permeability during each study visit. There was no evidence of changes in intestinal permeability as the circulating concentrations of LPS did not change over the follow-up period (Table 6).

### 4.7. Gut Microbiome

**Figure 2:**
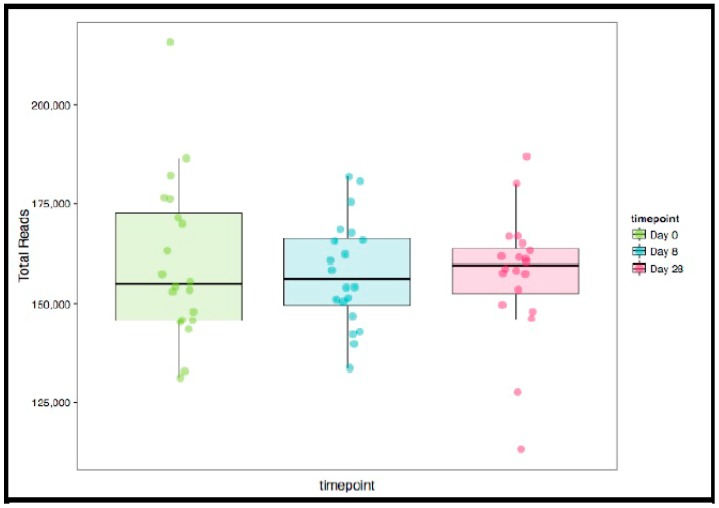
Box and whisker plots of sample library size with each point representing the number of reads in the sample in the three groups (Day 0, 8, 28). No significant differences were observed in any of the variables in the three time points evaluated (ANOVA for repeated measures).

**Figure 3:**
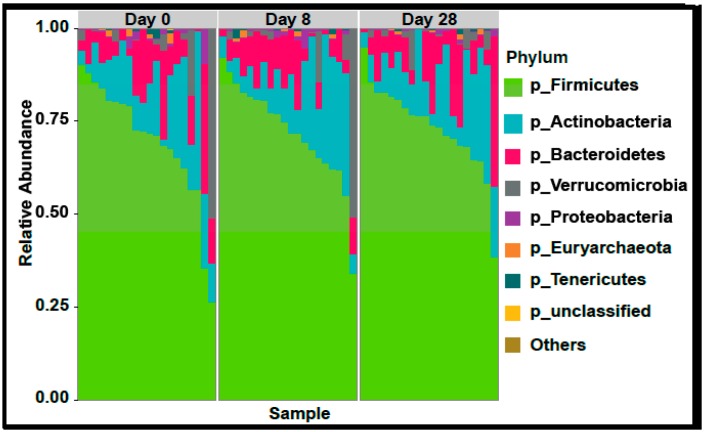
Plots showing the most abundant taxa at the phylum level as proportional abundances in the three groups (Day 0, 8, 28). No significant differences were observed in any of the variables in the three time points evaluated (ANOVA for repeated measures).

**Figure 4:**
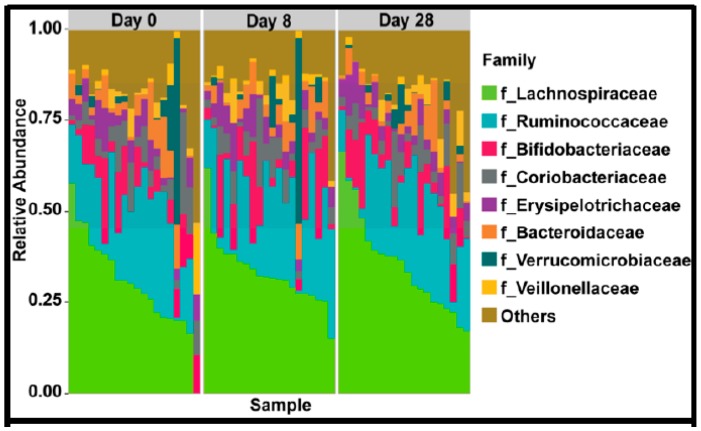
Plots showing the most abundant taxa at the family level as proportional abundances in the three groups (Day 0, 8, 28).

In the 9,499,927 reads, 1185 OTUs were observed following independent filtering. Multigroup analysis (PERMANOVA, *p*-value: 0.05) revealed no significant differences in microbiome alpha and beta diversity (Figure 2). Differential feature selection analysis revealed no significantly different OTUs throughout the time course (D0, D8 and D28). The eight most abundant phyla within sample microbiome are shown in Figure 3 and the eight most abundant families within sample microbiomes are shown in Figure 4, and data are shown in Supplementary Tables S1 and S2.

### 4.8. GI Symptoms in Response to HA35

There were no serious adverse events in any subject. No subject experienced severe bloating, cramping, abdominal pain, constipation or diarrhea over the follow-up period. One patient experienced severe flatulence for several hours after the first day of HA35 administration. This resolved in 2 days. No other patients experienced flatulence. In addition, one patient experienced severe nausea (without vomiting) that lasted for several hours. No other patients experienced nausea at any severity. The remainder of the side effects where either mild or moderate and resolved within several hours. Several subjects reported that their symptoms could have been from foods they ate rather than the HA35 itself (Table 7).

## 5. Discussion

To the best of our knowledge, this Food and Drug Administration approved longitudinal trial is the first study of oral HA35 administered to healthy human subjects for determining its safety and tolerability. Our observations agree with that of previous human studies of oral administration of similar doses of large molecular weight HA for joint pain, which also showed no adverse effects to the subjects [32,33]. The doses chosen for administration were double those effective in mouse models, and calculated based on scaling calculations of mice to man drug delivery based on intestinal surface area ratio calculations [14] HA35 was well tolerated with no significant adverse effects. No changes in biochemical or whole body metabolism were observed. Interestingly, in healthy subjects, there were no differences in either systemic inflammation or measures of intestinal permeability. These data show that HA35 can be used safely in humans in doses that may provide significant gut protection and systemic anti-inflammatory responses as observed in preclinical models.

Hyaluronan has systemic effects due to the presence of HA receptors in multiple organs [1,3]. Our studies showed that no adverse effects were noted in any of the organs evaluated. None of the subjects had elevated C reactive protein, a non-specific indicator of systemic inflammation and cardiovascular morbidity at baseline or in response to HA35 during follow-up. We also measured serum IL6, a cytokine produced by a number of immune cells, including macrophages and T cells, as well as epithelial and endothelial cells, in response to microbial infection or in response to other cytokines, such as TNFα. Concentrations of IL6 and TNFα are typically elevated during infections and sepsis [34]. HA35 did not change circulating IL6 or TNFα concentrations, suggesting that HA35 did not adversely impact proinflammatory cytokine profiles in healthy subjects. Of note, while HA35 did not have adverse effects on pro-inflammatory cytokines, the treatment also did not lead to a reduction in serum CRP, IL6 or TNFα in healthy subjects. This lack of reduction in inflammatory markers may not necessarily indicate a lack of anti-inflammatory effects of HA35, since the baseline levels of cytokines were already low in these healthy subjects. It is possible that HA35 could alter the circulating HA fragment distribution towards an anti-inflammatory profile by altering either HAS or hyaluronidases and would be considered in future studies.

One potential mechanism of benefit of HA35 is via regulation of gut permeability [16]. Gut inflammatory cell infiltration is a potential factor that contributes to increased gut permeability. We quantified fecal calprotectin, a calcium and zinc binding protein released from neutrophils and monocytes, that is used as a measure of intestinal inflammation [19]. HA35 did not change the fecal content of calprotectin. Human β-defensin 2 levels in stool, a component of the innate defense system in the gastrointestinal tract and an antimicrobial protein induced in response to microorganisms and proinflammatory cytokines [18,20], was also not affected by oral HA35 in healthy subjects. Since both fecal calprotectin and human β-defensin 2 are secreted proteins, we normalized them to total fecal protein and did not observe any difference in absolute or normalized values, reiterating our interpretation that gut inflammation and secretion of proteins were unaltered by HA35. These data are also consistent with the low concentrations of circulating LPS, an indicator of gut permeability, both at baseline and after oral HA35 supplementation.

The gut microbiome is increasingly recognized to contribute to perturbations in tissue responses and injury. Our analysis showed no differences in the stool microbiome diversity, or abundance of specific bacteria before, during and after a washout period of HA suggesting that HA35 does not alter the stool microbiome in healthy subjects over the period of time it was administered. Our studies after the washout period also show no changes in the stool microbiome. Even though it is possible that the overnight fast in these subjects may have potentially impacted the fecal microbiome, it is unlikely that this brief duration of starvation would have significantly impacted our observations. None of the subjects had any serious adverse events during or after the HA35 supplementation. Minor gastrointestinal symptoms, including transient bloating and cramping were reported in a minority of subjects, but were felt to be similar to their symptoms prior to inclusion in the study and had the same frequency after discontinuation of the supplement suggesting an excellent tolerance of HA35. We specifically chose young healthy volunteers for the safety study to avoid underlying undiagnosed conditions that become more frequent with age, however we do not anticipate any differences in the safety of HA35 if administered beyond the age range studied. However, answering this question will require studies specifically in subjects at extremes of age.

These observations demonstrate that HA35 is well tolerated and does not cause systemic or intestinal perturbations in healthy human subjects in the doses evaluated. There were no gender-based differences in our observations. HA35 offers a promising option to improve gut inflammation and potentially other systemic inflammatory disorders.

## Figures and Tables

**Table 1:** Anthropometric and clinical characteristics of subjects.

Anthropometric Data	Day 0	Day 8	Day 28
Number	20	20	20
Height (meters)	1.7 ± 0.1 (1.5–1.9)	1.7 ± 0.1 (1.5–1.9)	1.7 ± 0.1 (1.5–1.9)
Weight (kilograms)	79.0 ± 16.3	78.7 ± 16.4	78.9 ± 16.9
SBP (mmHg)	120.9 ± 15.3	121.3 ± 11.0	120.5 ± 11.8
DBP (mmHg)	74.4 ± 10.6	70.6 ± 11	71.5 ± 10.3
BMI (kg/m^2^)	27.4 ± 5.4	27.2 ± 5.5	27.2 ± 5.7
HR (beats per minute)	76.2 ± 13.6	73.9 ± 11.1	75.7 ± 10.2

BMI, body mass index; DBP, diastolic blood pressure; SBP, systolic blood pressure. No significant differences were observed in any of the variables in the three time points evaluated (ANOVA for repeated measures).

**Table 2:** Clinical laboratory parameters.

Parameter	Day 0	Day 8	Day 28
Number	20	20	20
Leukocyte count k/μL (3.7–11)	6.0 ± 1.6	6.1 ± 1.4	6.2 ± 1.5
Hemoglobin g/dL (13–17)	13.4 ± 1.5	13.4 ± 1.4	13.2 ± 1.5
Platelet Count k/μL (150–400)	263 ± 59	262 ± 63	271 ± 64
Protein g/dL (6.0–8.4)	7.4 ± 0.3	7.3 ± 0.4	7.3 ± 0.4
Albumin g/dL (3.5–5.0)	4.4 ± 0.3	4.4 ± 0.3	4.3 ± 0.3
Plasma calcium mg/dL (8.5–10.5)	9.2 ± 0.4	9.2 ± 0.2	9.1 ± 0.3
Total bilirubin mg/dL (0.0–1.5)	0.4 ± 0.3	0.4 ± 0.2	0.4 ± 0.3
Alkaline phosphatase U/L (40–150)	66 ± 16	66 ± 17	67 ± 17
AST U/L (7–40)	18 ± 6	22 ± 18	19 ± 7
ALT U/L (5–50)	16 ± 7	15 ± 6	18 ± 17
Glucose mg/dL (60–100)	92 ± 20	94 ± 16	89 ± 27
BUN mg/dL (10–25)	11 ± 3	11 ± 4	12 ± 3
Creatinine mg/dL (70–1.40)	0.85 ± 0.16	0.85 ± 0.16	0.83 ± 0.15
Sodium mmol/L (135–146)	140 ± 1	140 ± 2	140 ± 2
Potassium mmol/L (3.5–5.0)	4.0 ± 0.2	4.1 ± 0.3	4.0 ± 0.2
Bicarbonate mmol/L (23–32)	25 ± 2	24 ± 2	24 ± 2

ALT alanine amino transferase; AST aspartate amino transferase; BUN blood urea nitrogen; All data mean ± SD. Biochemical values in serum unless specified; values in parentheses are normal range for the clinical chemistry laboratory. No significant differences were observed in any of the variables in the three time points evaluated (ANOVA for repeated measures).

**Table 3:** Indirect calorimetry data.

Measurement	Day 0	Day 8	Day 28
Number	20	20	20
V02 (L/min)	0.23 ± 0.04	0.22 ± 0.04	0.24 ± 0.05
VC02 (L/min)	0.18 ± 0.03	0.18 ± 0.04	0.20 ± 0.04
Respiratory quotient	0.81 ± 0.08	0.82 ± 0.06	0.83 ± 0.071
Measured REE (Kcal/day)	1556.3 ± 274.5	1562.8 ± 221.4	1670.6 ± 333.9
Metabolic Rate (Kcal/day)	1653.1 ± 268.7	1665.2 ± 258.8	1668.4 ± 259.8
REE (% predicted)	95.9 ± 9.5	95.7 ± 12.9	101.4 ± 10.4

REE, resting energy expenditure; All data as mean ± SD. No significant differences were observed in any of the variables in the three time points evaluated (ANOVA for repeated measures).

**Table 4:** Serum Indicators of Inflammation and Injury.

Protein	Day 0	Day 8	Day 28
Number	20	20	20
TNF alpha (pg/mL)	2.0 ± 5.1	2.4 ± 6.6	3.2 ± 6.9
IL-6 (pg/mL)	9.6 ± 14.6	11.7 ± 16.8	10.5 ± 14.6
C- Reactive Protein (g/dL)	10.2 ± 15.5	10.3 ±12.8	11.6 ± 13.7

No significant differences were observed in any of the variables in the three time points evaluated (ANOVA for repeated measures).

**Table 5:** Fecal Peptides and soluble protein.

Fecal Proteins	Day 0	Day 8	Day 28
Number	20	20	20
Total soluble fecal protein (μg/mL)	22,883.8 ± 6425.7	22,365.3 ± 5872.8	22,726.3 ± 6305.2
Calprotectin (ng/mL)	139.3 ± 89.1	177.4 ± 133.1	195.00 ± 117.0
Normalized calprotectin (ng/μg protein)	0.006 ± 0.003	0.008 ± 0.006	0.009 ± 0.006
Human β-defensin 2 (ng/mL)	74.13 ± 109.0	92.06 ± 168.9	67.48 ± 116.5
Normalized β-defensin 2 (ng/μg protein)	0.004 ± 0.007	0.005 ± 0.0105	0.004 ± 0.008

No significant differences were observed in any of the variables in the three time points evaluated (ANOVA for repeated measures).

**Table 6:** Serum measures of LPS and hyaluronan (HA).

Measurement	Day 0	Day 8	Day 28
Number	20	20	20
Serum HA (ng/mL)	338.8 ± 82.8	329.0 ± 106.6	338.7 ± 73.9
LPS (ng/mL)	0.41 ± 1.35	2.68 ± 5.96	1.26 ± 4.03

LPS lipopolysaccharide; HA hyaluronan. No significant differences were observed in any of the variables in the three time points evaluated (ANOVA for repeated measures).

**Table 7:** Symptoms in subjects treated with HA35.

Symptom	Number of Patients Experiencing Symptoms (%)	Severity of Symptoms (%)	Frequency of Symptoms (%)
Bloating	3 (15)	None: 17 (85)	3 (15) several hours
		Mild: 2 (10)	
		Moderate 1 (5)	
Cramping	8 (40)	None: 12 (60)	8 (40) several hours
		Mild: 2 (10)	
		Moderate 6 (30)	
Nausea	1 (5)	None: 19 (95)	1 (5) several hours
		Severe: 1 (5)	
Increased Hunger	1 (5)	None: 19 (95)	1 (5) several hours
		Mild: 1 (5)	
Flatulence	1 (5)	None: 19 (95)	1 (5) more than one day
		Severe: 1 (5)	
Abdominal Pain	1 (5)	None: 19 (95)	1 (5) several hours
		Moderate: 1 (5)	
Constipation	1 (5)	None: 19 (95)	1 (5) several hours
		Moderate: 1 (5)	
Diarrhea	1 (5)	None: 19 (95)	1 (5) several hours
		Moderate: 1 (5)	

## Supplementary Materials

The following are available online at https://www.mdpi.com/2072-6643/11/5/1135/s1, Table S1. Composition at the Phylum Level, Table S2. Composition at the Family Level.
