# Supplementary material for: Safety of Hyaluronan 35 in Healthy Human Subjects: A Pilot Study
Source: Nutrients. 2019 May 22;11(5):1135. doi: 10.3390/nu11051135 (PMC6566413; doi:10.3390/nu11051135)
Supplement: Supplementary file 1 [file nutrients-11-01135-s001.pdf]

Supplementary Table S1. Composition at the Phylum Level

**Phylum**	**Day 0**	**Day 8**	Day 28
Firmicutes	0.697 (0.164)	0.722 (0.133)	0.735 (0.118)
Actinobacteria	0.146 (0.0985)	0.145 (0.111)	0.141 (0.0951)
Bacteroidetes	0.0969 (0.0855)	0.0789 (0.0497)	0.101 (0.102)
Verrucomicrobia	0.0415 (0.117)	0.0398 (0.115)	0.0151 (0.0273)
Proteobacteria	0.0111 (0.0211)	0.00789 (0.0118)	0.00432 (0.00509)
Euryarchaeota	0.00381 (0.00797)	0.00295 (0.00558)	0.00221 (0.00463)
Tenericutes	0.00275 (0.00605)	0.00267 (0.00602)	0.00152 (0.00322)
Unclassified	0.000272 (6.33e-05)	0.000243 (6.18e-05)	0.000237 (7.33e-05)
Others	0.000359 (0.000589)	0.000134 (0.000247)	0.000183 (0.00031)

Supplementary Table S2. Composition at the Family Level

**Family**	**Day 0**	**Day 8**	Day 28
Lachnospiraceae	0.3 (0.13)	0.331 (0.0926)	0.354 (0.135)
Ruminococcaceae	0.204 (0.104)	0.212 (0.113)	0.193 (0.112)
Bifidobacteriaceae	0.0772 (0.0625)	0.0864 (0.095)	0.0815 (0.0681)
Coriobacteriaceae	0.0679 (0.064)	0.0583 (0.0523)	0.059 (0.0517)
Erysipelotrichaceae	0.0504 (0.0323)	0.0503 (0.0373)	0.0481 (0.0315)
Bacteroidaceae	0.0504 (0.0507)	0.0454 (0.0377)	0.0379 (0.0485)
Verrucomicrobiaceae	0.0415 (0.117)	0.0398 (0.115)	0.0151 (0.0273)
Veillonellaceae	0.0301 (0.0447)	0.03 (0.0337)	0.0288 (0.0287)
Others	0.178 (0.103)	0.147 (0.075)	0.182 (0.107)
